# Endothelial properties of third-trimester amniotic fluid stem cells cultured in hypoxia

**DOI:** 10.1186/s13287-015-0204-0

**Published:** 2015-10-31

**Authors:** Andrea Alex Schiavo, Chiara Franzin, Mattia Albiero, Martina Piccoli, Giovanna Spiro, Enrica Bertin, Luca Urbani, Silvia Visentin, Erich Cosmi, Gian Paolo Fadini, Paolo De Coppi, Michela Pozzobon

**Affiliations:** Stem Cells and Regenerative Medicine Laboratory, Foundation Institute of Pediatric Research Città della Speranza, Corso Stati Uniti 4, 35127 Padova, Italy; Department of Woman and Children Health, University of Padova, via Giustinani 2, 35100 Padova, Italy; Venetian Institute of Molecular Medicine, via Orus 2, 35129 Padova, Italy; Medicine Department (DIMED), University of Padova, via Giustiniani 2, 35100 Padova, Italy; Stem Cells and Regenerative Medicine Section, Developmental biology and Cancer Program, Institute of Child Health, University College London, 30 Guilford Street, WC1N 1EH London, UK

**Keywords:** c-Kit, Fetal stem cells, Tissue regeneration, Endothelial dysfunction, Ischemia

## Abstract

**Introduction:**

Endothelial dysfunction is found in different pathologies such as diabetes and renal and heart diseases, representing one of the major health problems. The reduced vasodilation of impaired endothelium starts a prothrombotic state associated with irregular blood flow. We aimed to explore the potential of amniotic fluid stem (AFS) cells as a source for regenerative medicine in this field; for the first time, we focused on third-trimester amniotic fluid AFS cells and compared them with the already-described AFS cells from the second trimester.

**Methods:**

Cells from the two trimesters were cultured, selected and expanded in normoxia (20 % oxygen) and hypoxia (5 % oxygen). Cells were analysed to compare markers, proliferation rate and differentiation abilities. Endothelial potential was assessed not only in vitro—Matrigel tube formation assay, acetylated human low-density lipoprotein (AcLDL) uptake—but also in vivo (Matrigel plug with cell injection and two animal models). Specifically, for the latter, we used established protocols to assess the involvement of AFS cells in two different mouse models of endothelial dysfunction: (1) a chronic ischemia model with local injection of cells and (2) an electric carotid damage where cells were systemically injected.

**Results:**

We isolated and expanded AFS cells from third-trimester amniotic fluid samples by using CD117 as a selection marker. Hypoxia enhanced the proliferation rate, the surface protein pattern was conserved between the trimesters and comparable differentiation was achieved after culture in both normoxia and hypoxia. Notably, the expression of early endothelial transcription factors and AngiomiRs was detected before and after induction. When in vivo, AFS cells from both trimesters expanded in hypoxia were able to rescue the surface blood flow when locally injected in mice after chronic ischemia damage, and importantly AFS cells at term of gestation possessed enhanced ability to fix carotid artery electric damage compared with AFS cells from the second trimester.

**Conclusions:**

To the best of our knowledge, this is the first research work that fully characterizes AFS cells from the third trimester for regenerative medicine purposes. The results highlight how AFS cells, in particular at term of gestation and cultured in hypoxia, can be considered a promising source of stem cells possessing significant endothelial regenerative potential.

**Electronic supplementary material:**

The online version of this article (doi:10.1186/s13287-015-0204-0) contains supplementary material, which is available to authorized users.

## Introduction

Hypertension, coronary artery disease, peripheral artery disease, and chronic renal failure are correlated with endothelial dysfunction and a pro-inflammatory state, the earliest events in the onset of atherosclerosis. The effects of the artery occlusion are one of the major health problems, particularly in the adults [1] while in children reconstruction after major oncological or vascular surgery remains a difficult task. In this scenario, one of the greatest challenges is to obtain endothelial cells from non-vascular cell sources and researchers pay much attention on the use of embryonic and fetal stem cells as source for cell therapy. Endothelial progenitor cells derived from induced pluripotent stem and embryonic stem cells [2, 3], Wharton Jelly and umbilical cord blood (CB) cells [2] have been recently employed to recover the endothelial function after ischemia, but restricted therapeutic use due to safety issues and weak differentiation efficiency is limiting the applications of these cells. A specific subset of amniotic fluid stem (AFS) cells presenting the surface antigen CD117 (or c-Kit, the receptor for the stem cell factor, or SCF) has been isolated, demonstrated to be broadly multipotent [4, 5], and can be reprogrammed without viral transduction [6]. Because of their fetal but non-embryonic origin, AFS cells overcome many ethical concerns; moreover, they are easy to obtain from routinely scheduled procedures during the second trimester of pregnancy (i.e., amniocentesis and amnioreduction) [7], retain immunoprivileged properties [8] and do not form teratoma once injected in vivo [4]. Although CD117^+^ cells represent about 1 % of the initial cellular fraction of the whole amniotic fluid (AF) [9], their specific identification and subsequent expansion ex vivo make them a suitable candidate for regenerative medicine. While second-trimester AF requires a safe but invasive procedure during pregnancy, third-trimester AF could be taken at birth during cesarean sections [10]. Until now, it is not clear yet whether AFS cells isolated by the surface marker c-Kit could be found at term, since third-trimester AF cells have been isolated by their adhesion properties, rather than specific marker selection or by clonal approach [11], showing, for instance, osteogenic potential after differentiation in defined medium (evaluated by gene transcription) [12]. Here, we focused in particular on endothelial commitment with AFS cells from the second and third trimesters to promote vascular repair and restore perfusion in ischemic tissues. Additionally, we wanted to explore whether by culturing AFS cells in hypoxia we could improve their culture conditions. It is well known that stem cells normally reside in hypoxic niches [13], so next to the usual cell cultures performed under atmospheric oxygen tension (20 %), there are now many protocols for cell expansion at low oxygen concentration [14, 15]. Indeed, with particular attention to a long-term culture, hypoxia inhibits senescence, increases the proliferation rate, influences the potential of differentiation and modulates the paracrine effects of stem cells, causing upregulation of various secreted factors [16].

It has been demonstrated that in vivo oxygen tension changes from the periphery to the centre of an organ, with a lower oxygen level where stemness has to be maintained [17]. Within this context, mimicking the natural environment, oxygen became an important factor for in vitro stem cell expansion and potency studies. In this study, we focused on characterizing and comparing AFS cells from the second and third trimesters, giving emphasis on methods of isolation and expansion, phenotypic profile and endothelial marker expression pattern under normoxic and hypoxic conditions. The endothelial commitment of AFS cells cultured in hypoxia was also tested in vivo both in a mouse model of ischemic damage and in a carotid injury model.

## Methods

### Samples

The samples were collected in compliance with the Helsinki Declaration. AF samples were obtained after approval of the local ethics committee (Azienda Ospedaliera of Padova Pr. N. 32887/451P) by two different gestational periods from donors who signed informed consent. Specifically, (i) second-trimester AF samples were collected during routine amniocentesis performed at mid-gestation (16–18 weeks) for genetic screening purposes. Each sample was harvested as leftover material from amniocentesis. Only healthy samples were used. (ii) Third-trimester AF derived from healthy women undergoing eligible cesarean section. AF from cesarean section was unused material for clinical purposes.

### Seeding and selection of AFS cells

Samples were processed in accordance with the previous described method [18]. Briefly, cells from AFs were seeded on glass coverslips, and adherent cells were then positively selected for CD117 by using the MACS CD117 MicroBead Kit from Miltenyi Biotec (Bergisch Gladbach, Germany). Cells were cultured by using reconstituted Chang Medium C Lyophilized (Irvine Scientific, Santa Ana, CA, USA) before selection and then in expansion medium consisting of minimum essential medium alpha (Gibco, now part of Thermo Fisher Scientific, Waltham, MA, USA) containing 20 % Chang medium, 15 % fetal bovine serum (Gibco), antibiotics and L-Glutamine (2 mM final). Cells were maintained under standard 20 % O_2_, 5 % CO_2_ and 95 % relative humidity, referred to as “normoxia”. Cells were also cultured under “hypoxia” at 5 % O_2_ by using a CO_2_/O_2_ controller connected to a hypoxic chamber (BioSpherix, Lacona, NY, USA).

### Proliferation curves and doubling time

Between 50 and 100 cells were seeded on 96-multiwell plates, and proliferating cells were evaluated after 1, 2, 3, 6, and 9 days in culture. Cells were fixed with paraformaldehyde (PFA) (Sigma-Aldrich, St. Louis, MO, USA) 4 % in phosphate-buffered saline (PBS), washed in PBS twice for 5 minutes, a brief permeabilization was carried out with Triton X-100 0.5 % in PBS and cell nuclei were stained with 4′,6-diamidino-2-phenylindole (DAPI) (Sigma-Aldrich, St Louis, MO, USA). Cell numbers were evaluated by counting the nuclei on a series of randomized fields for the different samples and for each time point.

### Flow cytometry

FACSCalibur™ and Accuri C6 (BD Biosciences, Franklin Lakes, NJ, USA) flow cytometers were used. Acquired data were analyzed by using FlowJo software (TreeStar Inc., now part of FlowJo LLC, Ashland, OR, USA) after gating on viable cells. Cells analyzed starting from freshly retrieved AF samples were filtered through 70-μm nylon mesh filter and then stained as described above. Fresh AF and expanded AFS cells after different passages in culture were analyzed with antibodies listed in Table 1.

**Table 1:** List of antibodies

Antibody	Conjugation	Dilution/procedure	Manufacturer
CD9	FITC		Biolegend
CD29	FITC		Biolegend
CD31	FITC		Biolegend
CD34	FITC		Miltenyi Biotec
CD44	FITC		eBioscience
CD45	FITC		BD Pharmingen
CD56	PE		Beckman Coulter
CD73	PE		Biolegend
CD90	FITC		Biolegend
CD105	PE		Beckman Coulter
CD117	APC		Miltenyi Biotec
CD146	FITC		BioCytex
CD166	PE		Beckman Coulter
CD184	PE		Beckman Coulter
CD271	PE		Miltenyi Biotec
HLA-ABC	FITC		Beckman Coulter
HLA-DR	PE		Beckman Coulter
vWF		1:100; 1 hour; 37 °C	Dako
Anti-human mitochondria		1:80; 1 hour; 37 °C	Abcam
Anti-mouse CD31		1:80; overnight; 37 °C	Millipore
Anti-human CD31		1:80; overnight; 37 °C	Chemicon Europe
SSEA4		1:100; 1 hour; 37 °C	Abcam
Chicken anti-rabbit	Alexafluor 488	1:150; 1 hour; 37 °C	Life Technologies
Goat anti-mouse	Alexafluor 594	1:150; 1 hour; 37 °C	Life Technologies
Hamster anti-mouse	Alexafluor 594	1:150; 1 hour; 37 °C	Life Technologies

*FITC* fluorescein isothiocyanate, *PE* phycoerythrin, *APC* allophycocyanin, *vWF* von Willebrand factor, *SSEA4* stage-specific embryonic antigen 4

### Cell cycle analysis

The staining solution consisted in PBS containing Triton X-100 (0.1 %; Fluka,), DNAse-free RNAse A (0.2 mg/ml; Sigma-Aldrich, St Louis, MO, USA) and propidium iodide (1 mg/ml; Sigma-Aldrich, St Louis, MO, USA). After resuspension in cold PBS and ethanol, tubes were stored at −20 °C for at least 24 hours. After staining with 300 μl/10^6^ cells of staining solution, cells were analyzed.

### In vitro endothelial differentiation

To test the endothelial potential of AFS cells, we used the endothelial cell tube formation assay [19] over Matrigel™ Basement Membrane Matrix (BD Biosciences, East Rutherford, NJ, USA). Human umbilical vein endothelial cells (HUVECs), kindly provided by Marina de Bernard (University of Padova), were cultured in endothelial medium (PromoCell, Heidelberg, Germany) and used just after passage 2. AFS cells and HUVECs were detached from the original expansion culture and seeded in EGM-2 (endothelial growth medium-2) medium (Lonza, Basel, Switzerland) at a concentration of 15,000 cells/cm^2^ over the solidified coating.

ImageJ software [20] coupled with Carpentier G. Angiogenesis Analyzer [21] was used. For immunostainings, cells were fixed with PFA 4 % in PBS and permeabilized with Triton X-100 0.5 % in PBS.

To test functionality [22], EGM-2 medium was replaced with fresh medium containing Alexa Fluor® 488 conjugated with 10 μg/ml acetylated human low-density lipoprotein (AcLDL) (Molecular Probes, now part of Thermo Fisher Scientific). After 6 hours, medium was removed, and cells were fixed with PFA 4 % for later observation. Cell nuclei were counterstained with DAPI.

### In vivo experiments

All the procedures involving animals and their care were conducted in accordance with international guidelines, with the National Institutes of Health Principles of Laboratory Animal Care (National Institutes of Health publication 85–23, revised 1985) and were also approved by the local ethics committee for animal care of the University of Padova (organismo per il benessere degli animali, or OPBA).

### Matrigel plug

BALB/c strain Rag2−/−γc−/− immunodeficient mice were used in order to avoid the possible cell rejection after xenotransplant; 1 × 10^5^ cells per plug were resuspended in 500 μl of Matrigel with 0.75 mg/ml heparin (Pharmatex Italia, Milan, Italy), 50 ng/ml mouse recombinant fibroblast growth factor-basic (PeproTech, Rocky Hill, NJ, USA) and 100 ng/ml human recombinant VEGF (PeproTech). Mice were anesthetized with isoflurane, and cold liquid Matrigel containing cells or PBS was injected in the back lumbar region. After 14 days, mice were euthanized and plugs were harvested to obtain cryosections or to measure hemoglobin (Hb).

### Ischemia model

BALB/c strain Rag2−/−γc−/− immunodeficient mice were subjected to hind-limb ischemia on the right and left hind limbs [23]. After anesthesia with xylazine (20 mg/kg) and ketamine (100 mg/kg) by intraperitoneal injection, the femoral artery was excised and cauterized from its proximal origin as a branch of the external iliac artery to the distal point where it bifurcates into the saphenous and popliteal arteries. Forty-eight hours after ischemic damage, 5 × 10^5^ AFS cells cultured at 5 % O_2_ from the second (four mice) or the third (four mice) trimester were injected in the adductor muscle of the right leg while the left leg was used as control and PBS was injected (four mice). Hind-limb microvascular perfusion was measured with the Periscan-Pim II Laser Doppler System (Perimed AB, Järfälla, Sweden) 15 days after surgery. Specifically, Perimed proprietary software was used to calculate the ischemic ratio by dividing the perfusion of the ischemic limb versus the controlateral limb. For this specific calculation, region of interest (ROI) was chosen including each hind limb (left and right, respectively) captured by the laser Doppler image. Each ROI was equal in size for right and left hind limbs (Additional file 1). Each measure was repeated five times.

### Electric injury model

In accordance with the procedure described in Brouchet et al. [24], the left common carotid artery was exposed via an anterior incision of the neck in 8-week-old BALB/c strain Rag2−/−γc−/− immunodeficient mice (four mice with PBS as control, four mice with AFS cells from the second trimester and four mice with AFS cells from the third trimester) weighting 20 g on average and under isofluorane anesthesia. The carotid artery was injured by electricity with a bipolar microregulator (Pfizer, New York, NY, USA), and a 2-W discharge was applied for 2 seconds to each millimeter of carotid artery over a total length of 4 mm with the help of a size marker placed parallel to the long axis of the carotid. Three days later, the endothelial regeneration process was evaluated by staining the denuded areas with Evans blue dye as previously described [25]. Briefly, anesthetized animals were systemically injected via tail vein with 500 ul of 1 % Evans blue in PBS. After 30 minutes, animals were euthanized and immediately perfused with 50 ml of PBS to remove excess of Evans blue. The left common carotid artery was dissected and placed between slides with mounting medium (Bioptica, Cambridge, UK). Pictures were acquired by using a Leica DMI 6000B inverted microscope (Leica, Wetzlar, Germany), and the ratio between the damaged area stained in blue and the total carotid artery area was calculated by using ImageJ software.

### Immunofluorescence

Matrigel plugs were fixed for 1–2 hours with PFA 4 % in PBS and then dehydration was performed by sucrose gradient method at 4 °C with sucrose 10 % in PBS for 1 hour, 15 % for 1 hour and 30 % overnight. Pieces were then frozen in liquid nitrogen. Muscle samples were frozen by submersion in isopentane cooled with liquid nitrogen. Cryosections were obtained after embedding samples in OCT (optimum cutting temperature) compound (Kaltek, Padua, Italy) and using a cryostat (Leica) to produce sections. For specific antibody concentrations and conditions, see Table 1. Phase-contrast and bright-field pictures were taken by using an Olympus IX71 inverted microscope (Olympus, Tokyo, Japan). Immunofluorescence pictures were acquired by using a Leica DMI 6000B inverted microscope.

### Hemoglobin quantifications

Matrigel plugs were smashed with liquid nitrogen and the resulting powder was put in distilled water. Cell residues were pelleted and supernatants were used for Hb quantification by using Drabkin’s Reagent (Sigma-Aldrich). Reaction product absorbance (or optical density) was read at 540 nm with a SpectraMax Plus Spectrophotometer (Molecular Devices). Hb values were normalized for the protein content of Matrigel plugs, obtained by using Bradford Reagent (Sigma-Aldrich) and in accordance with the instructions of the manufacturer. Briefly, after incubation of an aliquot of lysate (the same used for Hb quantification) with the reagent and incubation for 30 minutes at room temperature, protein absorbance was read at 595 nm.

### Total RNA extraction and reverse transcription

Total RNA, including small non-coding RNA, was extracted by using miRNeasy Mini Kit (Qiagen, Venlo, The Netherlands) in accordance with the instructions of the manufacturer. RNA was quantified by using Nanodrop2000 (Thermo Fisher Scientific), and 1 μg was retrotranscribed with miScript II RT kit (Qiagen) by using HiFlex buffer that allows the further quantification of both mature miRNA and mRNA. All procedures were carried out by following the instructions of the manufacturer and using GeneAmp® 2720 thermal cycler (Applied Biosystems, Waltham, MA, USA).

### Real-time polymerase chain reaction

For mRNA analysis, quantitative real-time polymerase chain reaction (qPCR) was performed by using Platinum® SYBR® Green qPCR SuperMix-UDG (Life Technologies, Carlsbad, CA, USA), 5 ng of cDNA and a 300 nM mix of specific forward and reverse primers (final concentrations). For miRNA quantification, qPCR was performed by using miScript SYBR® Green PCR Kit (Qiagen), 10 ng of cDNA and specific miScript Primer Assays (Qiagen). Reactions were carried out in a LightCycler II instrument (Roche, Basel, Switzerland). Results are expressed in arbitrary units (A.U.) considering the ratio targetRNA/referenceRNA content (reference for mRNA: *β2MICROGLOBULIN*, abbreviated “*B2M*”; reference for miRNA: RNU6-2). For the mRNA and miRNA primer sequence list, see Tables 2 and 3.

**Table 2:** Primer sequences used for mRNA quantification

Gene	NM_	Forward primer	Reverse primer	Amplicon, base pairs
*ETV2*	014209.2	AGTCGGACCGTGCCAGTTTG	GAGCCACCTCTTTGGGGTCG	168
*FLI1*	002017.4	*GAGGAGCTTGGGGCAATAAC	*AGAGCAGCTCCAGGAGGAAT	195
*VEGF-A*	all isoforms	TCACCATGCAGATTATGCGGA	TGTTGTGCTGTAGGAAGCTCA	75
*B2M*	004048.2	CAACTTCAATGTCGGATGGATG	GCTGTGCTCGCGCTACTCT	161

*[28]

**Table 3:** Qiagen primer assays used for miRNA quantification

Assay number	Official symbol	Qiagen catalog number	Lot number
Hs_miR-126_1	MIR126	MS00003430	116330116
Hs_miR-132_1	MIR132	MS00003458	116330117
Hs_miR-210_1	MIR210	MS00003801	116330113
Hs_miR-221_1	MIR221	MS00003857	116330111
Hs_miR-222_2	MIR222	MS00007609	116330110
Hs_RNU6-2_11	N/A	MS00033740	117836183

### Statistical analysis

Data are expressed as standard error of the mean, and statistical comparisons were performed by using Student’s *t* test or one-way analysis of variance, as appropriate. Post-hoc Bonferroni’s correction for multiple comparisons was used. All *P* values of not more than 0.05 were considered statistically significant.

## Results

### Antigen expression of fresh AFS cells from second and third trimesters

**Fig. 1:**
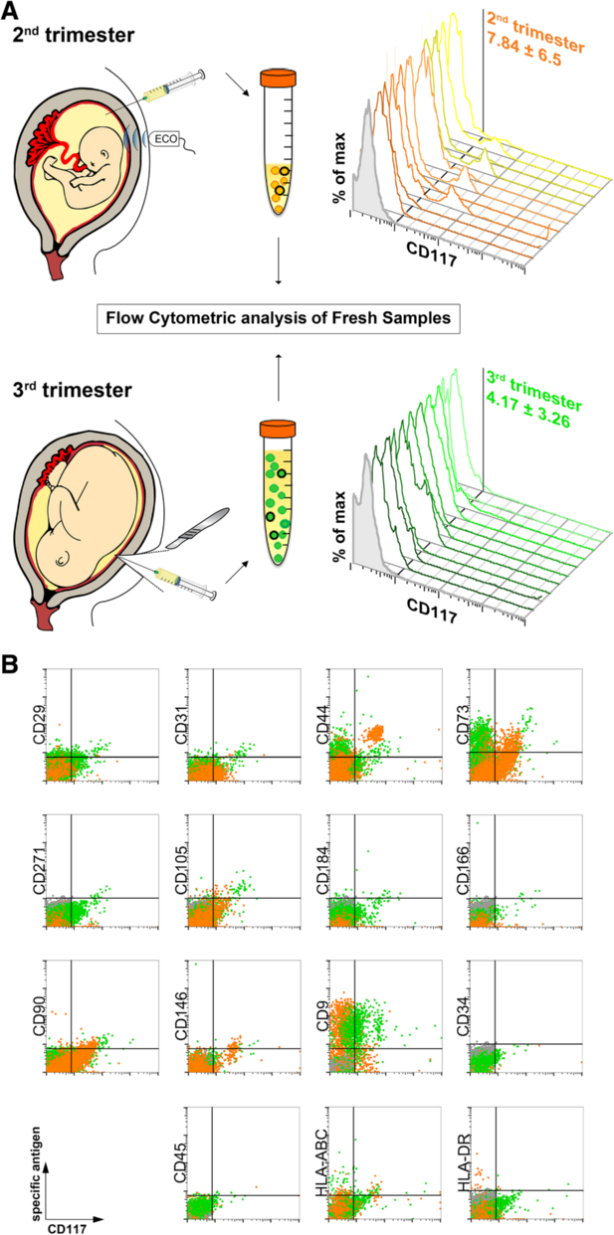
Cell isolation from collected amniotic fluid (from the second and third trimesters) and characterization by flow cytometry analysis. **a** Representative scheme of amniotic fluid retrieval for cell extraction from second-trimester amniocentesis (*upper part*) and cesarean section at term of pregnancy (*below*). Flow cytometry phenotyping revealed the variable presence of CD117 marker on freshly isolated cells (three-dimensional histograms, n = 10, isotype control in *gray*). **b** Representative dot-plot cytograms showing cells from both trimesters (second trimester in *orange* and third trimester in *green*) comparing various antigens (*vertical axes*) versus CD117 expression (*horizontal axes*)

The phenotypic characterization of freshly isolated cells from both trimesters revealed high variability on the presence of CD117^+^ cells, and some samples possess a high portion of CD117^+^ cells and this was noticed for both the trimesters (7.84 ± 6.50 % and 4.17 ± 3.26 % for the second and third trimester, respectively; Fig. 1a); this is due to the intrinsic variability among samples. In keeping with other studies on CD117^+^ cells, cells from the second or third trimester were negative for the hematopoietic markers CD34 and CD45 and positive for the mesenchymal molecules CD73 (5′-nucleotidase), CD44 (a receptor for hyaluronic acid and others components of extracellular matrices), CD105 (endoglin type I glycoprotein), CD90 (also called Thy-1) and CD146, a cell adhesion molecule also marking the endothelial lineage (Fig. 1b). In particular, in the third trimester, we detected only a small portion of CD117^+^ CD90^+^ cells while CD117^+^ CD105^+^ cells were more abundant. This difference in antigen expression was not detected in expanded cells. The fresh CD117^+^ fraction did not co-express molecules of the major histocompatibility complex type II (specifically HLA-DR), whereas the major histocompatibility complex type I (i.e., HLA-ABC) was present. The surface antigen CD9 was markedly detected in different proportion in the two trimesters: it was found exclusively on the CD117^−^ fraction of the second trimester, and it was detected in almost all CD117^+^ cells of the third trimester.

### Characterization of AFS cells from second and third trimesters expanded in normoxia and hypoxia

**Fig. 2:**
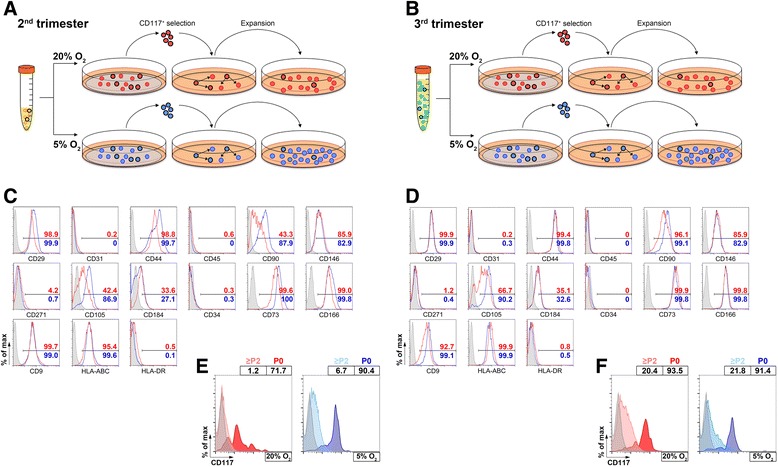
Selection, expansion of amniotic fluid stem cells and characterization of obtained cultures. **a**, **b** Cells contained in amniotic fluid of both trimesters were seeded over coverslips and then selected for CD117 antigen for subsequent expansion in culture, and two different oxygen concentrations were used for the experiments (20 % and 5 %). The obtained cells were then characterized for surface markers; here are representative data for the cells obtained from the second (**c**) and the third (**d**) trimester with their percentage of positive cells for each specific marker: *red lines* and numbers = normoxic condition (20 % O_2_); *blue lines* and numbers = hypoxic condition (5 % O_2_). The presence of the initial marker for selection (CD117) was monitored during the different passages in culture for second-trimester (**e**) and third-trimester (**f**) cells

**Fig. 3:**
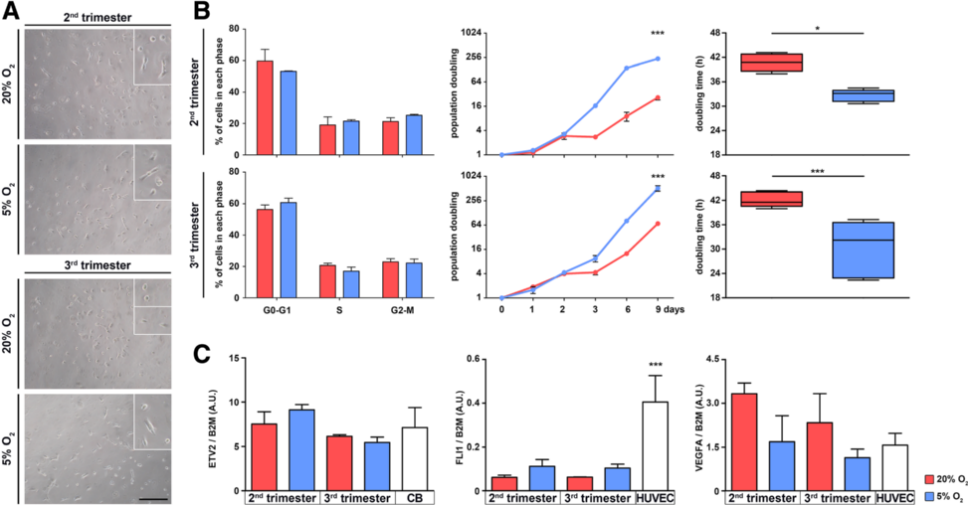
AFS cell morphology and behavior in culture. **a** AFS cells from both trimesters were expanded in normoxic (20 % O_2_) or hypoxic (5 % O_2_) conditions maintaining sub-confluence between passages (scale bar = 200 μm). **b** For each type of sample and culture condition, the cell cycle phases (G_0_-G_1_, S and G_2_-M) were quantified by flow cytometry after propidium iodide staining of the cells (*left graphs*). Cells in well were counted at different time points to obtain a growth curve (*central graphs*) and were expressed by population doublings at each measured point (n = 5). From the growth curve, the mean doubling time (*right graphs*) for each condition was calculated and compared to see whether hypoxia was influencing the time window of cell replication. **c** Endothelial transcription factor detection: *ETV2*, *FLI1* and *VEGFA* were expressed at basal level in AFS cells. *P < 0.05, ***P < 0.001. CB was used as positive control for ETV2 expression, whereas the adult HUVECs were used as positive control for the other two genes (*white bars*); 20 % O_2_ condition represented by *red bars and lines*; 5 % O_2_ condition represented by *blue bars and lines. AFS* amniotic fluid stem, *A.U.* arbitrary units, *CB* cord blood, *HUVEC* human umbilical vein endothelial cell

Cells yielded from third-trimester AF samples were usually higher in terms of number of cells per milliliter and had a more heterogeneous morphology after seeding when compared with the second trimester. However, we were able to obtain adherent c-Kit^+^ colonies with cells proliferating for several passages from cells derived from both trimester samples in normoxic and hypoxic conditions (Figs. 2a, b and 3a).

Similarly to what was observed before, the expression of c-Kit decreased after some passages in culture [8]. Importantly, there were some differences between the cultured cells, as shown in Fig. 2e. In second-trimester samples, there was a greater percentage of cells expressing CD117 after expansion in hypoxia rather than in normoxia, whereas third-trimester AFS cells (Fig. 2f) displayed a similar percentage of CD117^+^ cells concerning the two oxygen tensions; notably, this percentage was considerably higher in the third than in the second-trimester cells.

Regarding other markers, as shown in Di Trapani et al. [26], on one hand, cells were negative for the low-affinity nerve growth factor receptor (LNGFR), referred to as CD271, a versatile marker used to select multipotent mesenchymal stem cells [27], CD31 for mature endothelium, CD45 and CD34; on the other hand, cells were positive for the adhesion molecules CD166 (activated leukocyte adhesion molecule, or ALCAM), CD184, CXCR4, receptor of the stromal derived factor-1, or SDF-1), CD146, CD29 and CD44. In addition, the specific stem cell antigen, stage-specific embryonic antigen 4 (SSEA-4), was significantly present at low oxygen in AFS cells at term (Additional file 2).

Cell cycle (G_0_ and G_1_, S or G_2_ and M) was similar independently from the trimester of origin or from the oxygen condition (Fig. 3b, left graphs) underling the good cell division ability of both fetal cells. On the other side, the proliferation rate was increased for cells grown in hypoxia: after culturing at 5 % O_2_, significantly more cells were obtained for both trimester samples compared with their counterpart cultured in normoxia (*P* < 0.001, with an increase up to seven times the total number of cells, Fig. 3b, central graphs). The estimated doubling time came to be significantly diminished from culture at 5 % O_2_ in comparison with 20 % O_2_ (*P* < 0.05 for the second trimester and *P* < 0.001 for the third trimester), whereas there was no difference on the doubling time between cells of different trimesters expanded at the same oxygen level (Fig. 3b, right graphs). The doubling time in normoxic environment was consistent with previously reported studies on AFS cells [4].

Cells from both trimesters were assessed for their osteogenic and adipogenic potential. AFS cells in all conditions were able to deposit calcium oxalate and to produce lipid vacuoles when induced to differentiate respectively in osteoblast-like and adipocyte-like cells (Additional file 3), confirming their ability to differentiate into mesenchymal-derived lineages.

To unravel whether AFS cells possessed a molecular signature toward endothelial differentiation, we quantified the expression of *ETV2*, *FLI1* and *ERG1*, members of the E-twenty six transformation (ETS) family of transcription factors, which direct angiogenesis and endothelial progression [28] from development to post-natal life. Notably, AFS cells at the basal state strongly expressed *ETV2*, which is the earliest ETS factor to be activated. This is consistent with their fetal origin since *ETV2* is present in endothelial precursors during development and normally absent in adult cells (Fig. 3c, *left*). *FLI1*, present in a more mature endothelial state, was also expressed, although at lower levels (Fig. 3c, *centre*), whereas *ERG1* was not detected. Moreover, *VEGF* transcript, an important endothelial marker, was also found in AFS cells before endothelial induction (Fig. 3c, *right*).

### In vitro and in vivo endothelial formation

**Fig. 4:**
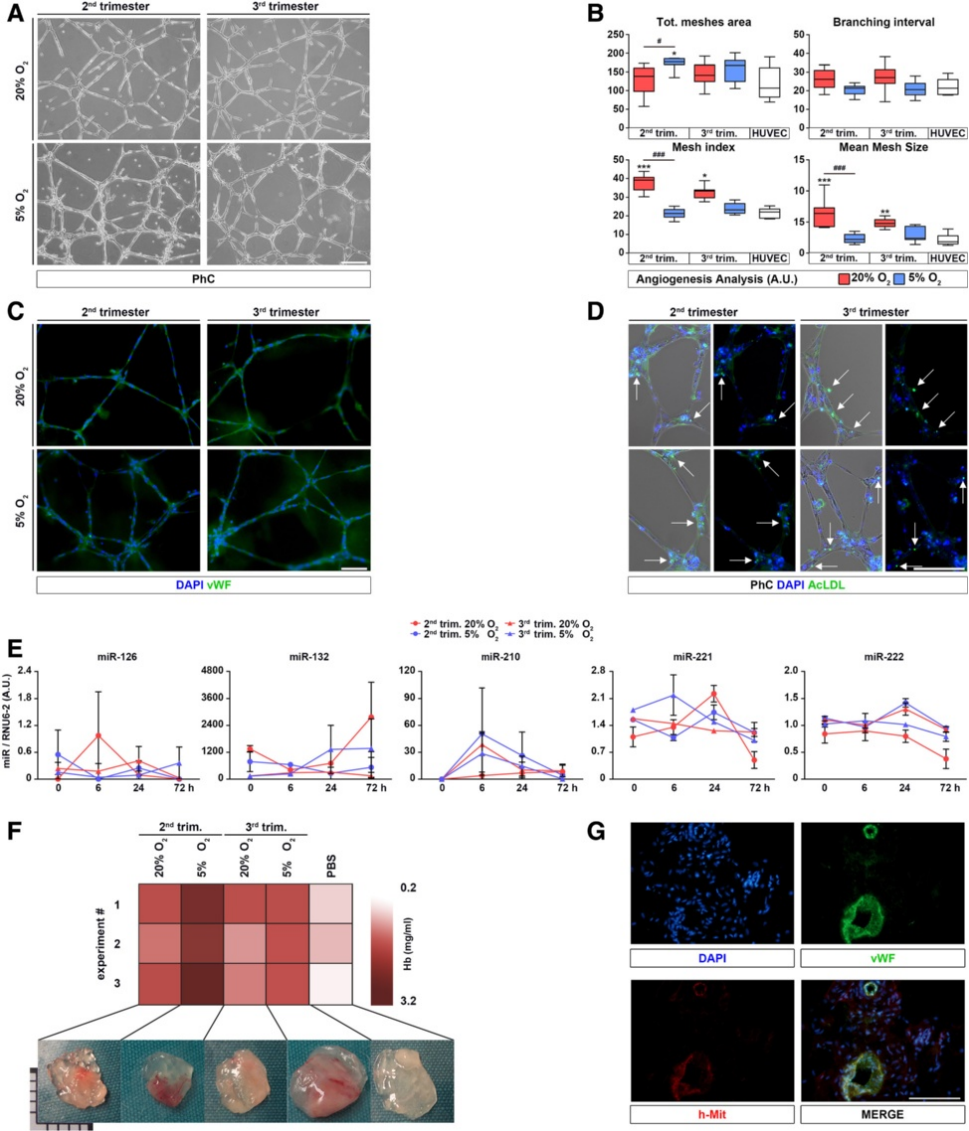
In vitro and in vivo endothelial differentiation and miR expression of AFS cells. **a** AFS cells were seeded over Matrigel-coated wells, and phase-contrast images showed network formation after 24 hours (scale bar = 100 μm). Image analysis of those network structures (**b**) produced values that were compared against standard positive control cells (HUVECs, *left white bars*), and statistical significance is shown among AFS cells and control with asterisks, also between different concentrations of oxygen for the same trimester with hashes (n = 10). Immunofluorescence stainings of endothelial network (**c**) showed vWF (green signal) expression (scale bar = 100 μm). **d** Ability of endothelial cells to intake AcLDL: green vesicles are indicated by *white arrows*, phase contrast (*left*) and fluorescence detection (*right*) are shown for better appreciation (scale bar = 200 μm). *P < 0.05, **P < 0.01, ***P < 0.001 ^#^ *P* < 0.05, ^###^ *P* < 0.001. **e** Time course of pro angiomiR (miR126, 132 and 210) and anti angiomiR (miR221 and 222) expression after differentiation. **f** *Upper row*: Matrigel plugs (n = 3 for each condition. Experiment # = experiment number) underwent hemoglobin (Hb) test: relative quantification of Hb was higher in plugs loaded with AFS cells cultured at 5 %. *Lower row*: appearance of the harvested plugs with and without cells before Hb quantification. **g** Representative expression of vWF (green signal) co-localized with a specific human antigen (human mitochondria, red signal) (scale bar = 50 μm). All nuclei in the figure were counterstained with DAPI. *AcLDL* acetylated-low density lipoprotein, *AFS* amniotic fluid stem, *A.U.* arbitrary units, *CB* cord blood, *DAPI* 4′,6-diamidino-2-phenylindole, *HUVEC* human umbilical vein endothelial cell, *PBS* phosphate-buffered saline, *PhC* phase contrast, *vWF* von Willebrand factor

Through the Matrigel tube formation assay, we were able to obtain successful formation of endothelial tubes mimicking capillary-like structures by using AFS cells from both trimesters after expansion in 20 % or 5 % O_2_ (Fig. 4a). The total area comprised in the meshes was similar in all conditions and comparable to that of HUVECs, used as a standard control. The branching interval was also equal among samples and conditions, indicating uniformity of structures and similarity in response to the endothelial cell tube formation assay. Mesh index and mean mesh size of AFS cells were similar to HUVECs or higher (Fig. 4b). Capillary-like structures expressed von Willebrand factor (vWF) in samples from both trimesters and cultured at both oxygen tensions (Fig. 4c), and the endothelial functionality was acquired as demonstrated by the uptake of the lipoprotein AcLDL (Fig. 4d). In addition, we analyzed the expression of AngiomiRs, specific miRNAs that can promote or block the angiogenic process. In particular, miR126, 132 and 210 are key positive regulators of angiogenesis and endothelial cell survival, whereas miR221 and 222 prevent endothelial cell migration [29]. miR126, 132, 221 and 222 were detectable in AFS cells at the basal state, whereas miR210, which was absent before angiogenic induction (Fig. 4e), was present already after 6 hours of endothelial differentiation and decreased 72 hours after endothelial stimulation in all the analyzed conditions. After 6, 24 and 72 hours of angiogenic induction, miR126 and 132 did not significantly change the expression level, as the anti-angiogenic miR221 and 222 were detectable in a quite stable trend.

Together with ETS factors and VEGF expression data, these results suggest that AFS cells are transcriptionally open for the angiogenic pathway; therefore, they may recognize and respond to proper stimuli and undergo endothelial differentiation. Lack of markedly different expression level between the two trimesters underlined the endothelial imprinting of the two cell types.

To confirm their propensity to generate functional vessels, AFS cells were also implanted in vivo, and neo-formed and perfused vessels were detected in Matrigel plug assay performed in immunodeficient mice (Fig. 4f). This macroscopic observation was further investigated by quantifying the Hb content in plugs from each condition in comparison with the relative controls. Every sample contained significantly higher levels of Hb with respect to controls while the optimal state was represented by the cells cultured in hypoxia (Fig. 4f, graph). In all of the different culture conditions, some vWF-expressing structures were also positive for the human mitochondria antigen (Fig. 4g) with the exception of control plugs where structures were positive only for the endothelial marker.

### Second- and third-trimester AFS cells regenerate damaged endothelium in vivo

To prove whether endothelium derived from AFS cells could function, two distinct animal models were used to mimic endothelial dysfunctions: ischemic damage after femoral excision and carotid injury. Cells cultured in hypoxia were chosen because of their superior capacity to proliferate.

**Fig. 5:**
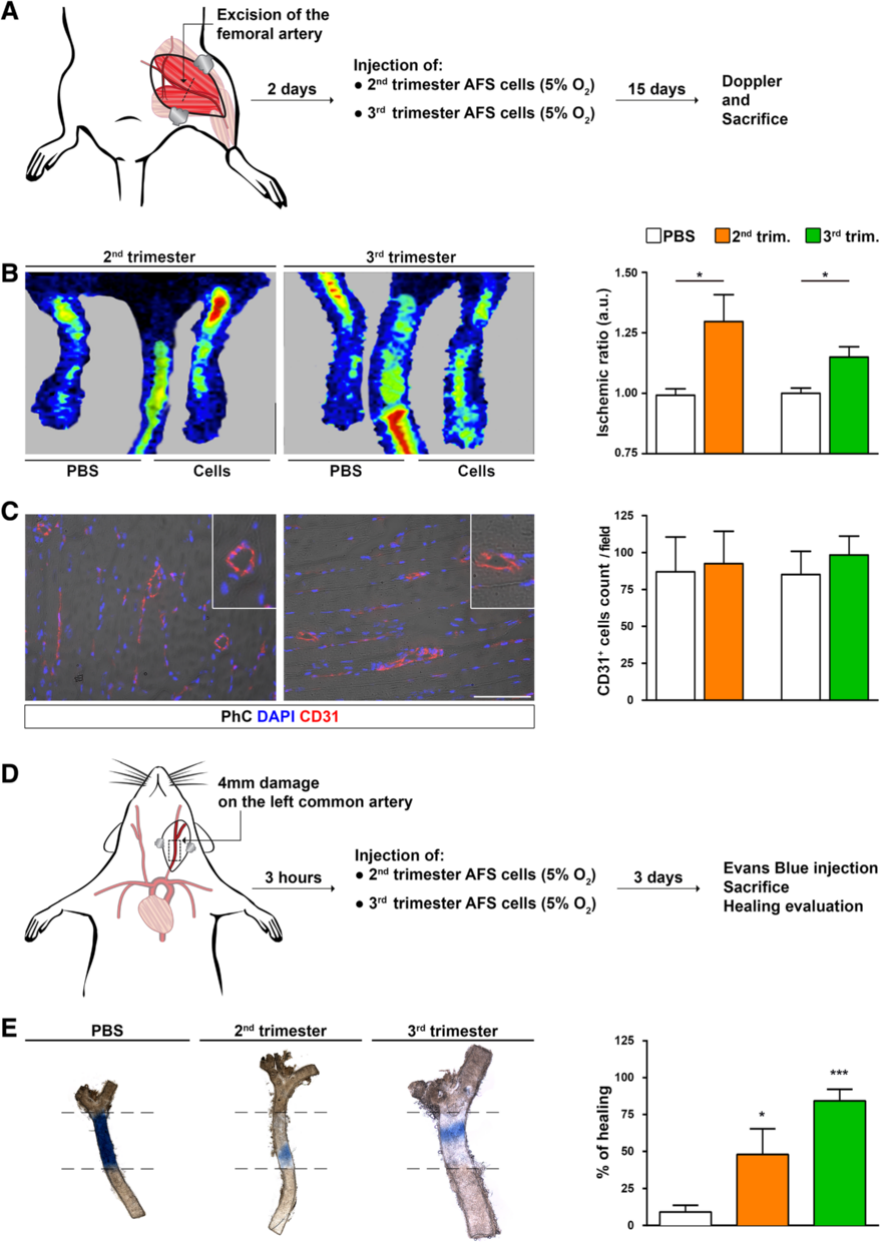
Endothelial rescue after AFS cell injection in vivo. **a** Experimental plan. Mouse model of acute ischemia: 48 hours after bilateral femoral vein excision, PBS or AFS cells from the second and third trimesters were injected. Fifteen days later, functional analysis and sacrifice have been performed. **b** In comparison of mice injected with PBS (control) and of those treated with cells, the ischemic ratio was significantly higher in the latter group (n = 4 for each condition). ROI used to calculate the ischemic ratio was equal in size for right and left limbs. **c** CD31 staining of cells in both control and treated animals (*left*) evidenced a positive trend in favour of the cell-treated mice but with no statistical differences (right) (n = 5 for each condition). **d** Experimental plan. Mouse model of carotid injury: 3 hours after damage, PBS or AFS cells cultured in hypoxia were injected. **e** Three days later, after Evans blue injection, it is possible to appreciate the quick recovery of the damaged endothelium after AFS cell injection. In particular, AFS cells of the third trimester proved to be significantly more effective than the cells from the second trimester (n = 3 for each condition). *P < 0.05, ***P < 0.001. *AFS* amniotic fluid stem, *DAPI* 4′,6-diamidino-2-phenylindole, *PBS* phosphate-buffered saline, *PhC* phase contrast

After the femoral artery excision, Doppler analysis evidenced how the superficial blood circulation was significantly ameliorated in cell-treated mice compared with PBS-injected muscles (Fig. 5b); an increased number of CD31^+^ capillaries was also observed in treated mice (double staining for anti-human and anti-murine CD31 was negative; data not shown). Although the difference in terms of CD31^+^ cells between PBS- and cell-injected muscles was not significant, the positive trend was noticeable after treatment with both second- and third-trimester AFS cells (Fig. 5c). By immunofluorescence for human antigens such as human Nuclei, anti-human Mitochondria and Lamin A/C, we tried to identify and confirm the participation of injected AFS cells in supporting angiogenesis and vascular regeneration in muscle, but very rare cells were found and no coexpression of endothelial marker was evident. Also, reverse transcription-PCR for human BM2 was performed without positive results (data not shown). Consequently, the endothelial recovery was postulated as outcome of a bystander effect. To study the indirect mechanism of endothelial reconstitution, a mouse model of electric carotid artery injury was subsequently used (Fig. 5d). After Evans blue injection, the impaired endothelium of control mice absorbed the blue staining while cell-treated animals underwent almost complete endothelial restoration 3 days after electric injury, as demonstrated by the very small presence of Evans blue-positive areas (Fig. 3e). In particular, AFS cells from the third trimester proved to be more effective than AFS cells from the second trimester in repairing the damaged artery. Given the endothelial wall of the cell-injected mice, it was clear how the shape was regained when compared with the loose endothelial wall of the PBS-treated animals.

## Discussion

In the present study, we compared the features of AFS cells from the third trimester with the ones of the well-defined second-trimester AFS cells. Specifically, we aimed to assess both in vitro and in vivo the differentiation potential toward endothelial lineage of third-trimester AFS cells under hypoxic conditions. Cells derived from AF at term have only recently been considered as an alternative source of stem cells [12, 30], and in particular this is the first study that looks at the c-Kit^+^ population derived at term for endothelial regeneration. The antigen profile gains in importance on the definition of both stemness and immunological properties. The latter has been recently addressed, showing that AFS cells from the second and third trimesters inhibit T and natural killer cells in a similar manner [26]. The evident overlapping of the characteristics of the two trimester sources has major implications since AFS cells from the last trimester of gestation are an easily available and abundant source without ethical concerns mainly because they are discarded material. It is possible that stemness maintenance during gestation is related to the low oxygen tension in the AF since oxygen concentration is a key element of both cell proliferation and differentiation [31]. In this regard, without the addition of synthetic chemicals and coating, 5 % oxygen turned out to be sufficient to halve the doubling time and to keep mostly unchanged both the phenotype and the differentiation ability in respect to 20 % of oxygen. For the latest point, our method of culture turned out to be of paramount importance: contrary to other researchers [32], we were able to confirm the endothelial differentiation comparable to HUVECs.

It is known that angiogenesis is a process starting from pre-existing vasculature and the first elements involved are endothelial cells that must activate to undergo proliferation, allowing the formation of new vessels. In our plug assay, AFS cells were able to switch from a stem to a more committed state and to build a functional endothelial network. In addition, the vessel formation inside the plug has been proven to be of human origin, further confirming that our cells actively contributed to the endothelial network.

Specific ETS transcription factors essential for angiogenic differentiation and vessel formation [28] are constitutively expressed by endothelial precursor cells. Following the work of Ginsberg et al., we focused our analysis on *ETV2*, present specifically in fetal endothelial precursors, *FLI1* and *ERG1*, detected in adult endothelial progenitor cells. Ginsberg et al. demonstrated how the insertion of the abovementioned genes in AF cells, depleted of c-Kit^+^ population, were able to easily convert AF cells into endothelial progenitor cells. Our results highlighted how c-Kit^+^ AFS cells are already transcriptionally open toward endothelial differentiation without the need of any genetic manipulation. *ETV2*, a marker of fetal endothelial precursor cells, was more present in AFS cells than in the CB cells used as control, whereas *FLI1* was detected at a lower level in respect to the control (HUVECs). This agrees both with the fetal origin of AFS cells and with their stemness state, but these cells also display an open state toward mature endothelium formation. It is possible that microRNAs (miRNAs) have a role in the post-trascriptional regulation of those genes [33, 34]. Specifically, we chose to look into the expression of miR126, 132 and 210, which promote angiogenesis by targeting negative regulators in angiogenic pathways. Endothelial cell-specific miR126 plays an essential role in neoangiogenesis following ischemia [35] and was detected in AFS cells before endothelial induction and maintained during differentiation. Importantly, the expression of miR210, which is a key regulator of angiogenesis [36], rapidly increased after induction. In addition, we focused on the anti-angiomiR221 and 222 [37], which inhibit the process of angiogenesis by degrading positive regulators. It has been demonstrated how these two miRNAs regulate the angiogenic activity of SCF and the level of its receptor c-Kit [38]. Our study underlines how AFS cells express also these anti-angiomiRs in the basal condition as if AFS cells could be ready to receive stimuli either in favor or against this endothelial differentiation. Overall, our in vitro results showed that AFS cells of the second and third trimesters both possess the gene expression profile for an efficient endothelial differentiation not only on the transcription factors side but also because the post-transcriptional modulators of angiogenic gene detection were revealed. Indeed it was evident that, although anti-angiomiRNA expression was maintained, cells in vitro were prone to differentiate toward endothelium, highlighting the ability to selectively receive and enhance the pro-angiogenic stimuli.

Different sources of mesenchymal stem cells of fetal and adult origin, from CB to adipose-derived cells or embryonic stem cells, have been shown to contribute to endothelial regeneration but the functionality of these cells has been only partially proven in vivo [2, 39–41] or are difficult to expand in culture [42]. In the well-established mouse model of ischemia used here, the cells were injected 48 hours after resection of both femoral arteries. In such a harsh microenvironment, the action of AFS cells of both trimesters was consistent and robust. They were indeed capable of ameliorating the blood flow as highlighted by Doppler analysis, although AFS cells were not detectable. This phenomenon is in keeping with previous literature describing that AFS cells exert an indirect effect through a paracrine action as demonstrated in the repair of intestine, kidney, lung and heart [43–46] and in a different model of endothelial damage [47]. In this ischemia model, AFS cells exert a bystander effect, but to deeply investigate this aspect toward endothelial regeneration, we decided to use a different animal model always based on artery damage. This specific model, defined by Carmeliet et al. [48] in the femoral artery and adapted by Brouchet et al. [24] for the carotid damage, well represents a model of indirect endothelial repair. Indeed, Hagensen et al. [49] investigated the mechanism of endothelial reconstitution, demonstrating how the model of carotid impairment itself regenerates spontaneously from the distal part to the centre of the damaged area. In particular, injured carotid from wild-type mouse transplanted into GFP animal proved how the activation of the resident healthy endothelium (GFP^+^ cells) toward the damaged area was the mechanism of the endothelium-restauration, giving up the theory that circulating endothelial progenitors cells could contribute themselves to de novo endothelial cells [50]. However, when bone marrow or endothelial cells are injected in the same mouse model, the ability to re-build the artery wall has been demonstrated to be superior and quicker [51, 52]. For these experimental reasons, we decided to perform AFS cell injection in damaged carotid, believing that cell contribution could improve the endothelial healing via the bystander effect. Indeed, 3 days after damage, the reduced area with Evans blue staining confirmed the attained regeneration. Given that this model regenerates spontaneously in 10 days [49], the synergy between AFS cells and the resident endothelium was clear since in control animals no endothelial restoring was seen after 3 days. In addition, we can appreciate the stronger ability of endothelial recovery of our cells with respect to HUVECs or bone marrow cells [51].

## Conclusions

In this work, we compared the features and the endothelial properties of AFS cells from two trimesters, characterized mainly by different accessibility and abundance. AFS cells from the third trimester, which have the advantage of being available from healthy fetuses of scheduled cesarean section, proved to possess comparable properties with respect to AFS cells from the earlier trimester but were characterized by a quicker proliferation rate when cultured in hypoxia. AFS cells of both trimesters were able to differentiate both in vitro and in vivo into endothelial cells and, in two different disease models, did sustain residing endothelial precursor cells to rescue tissue function, with the third-trimester cells demonstrating a stronger action in the carotid mouse model.

This is the first study that deeply characterizes the third-trimester AFS cells and investigates them as a new source for potential therapeutic applications. In the future, ex vivo engineering of endothelial structure using AFS cells will completely address the differentiation potential of AFS cells that so far did not directly generate new endothelium. Comparison of endothelial cells derived by direct differentiation or reprogramming could become necessary in order to decide the most efficient and safe method for their clinical use.

## Additional files

Additional file 1:
**Region of interest (ROI) visualization.** Screen shot showing a representative image of an analyzed animal after ischemic damage and cell injection. The ischemic ratio was calculated by using ROI of the same size for the right and left limbs (ROI1 and ROI2). (JPEG 897 kb) Additional file 2:
**Cell characterization.** (A) SSEA-4 staining for AFS cells in all culture conditions. (B) Percentage of SSEA-4-positive AFS cells in all conditions. *AFS* amniotic fluid stem, *SSEA-4* stage-specific embryonic antigen-4 (JPEG 2556 kb) Additional file 3:
**Adipogenic and osteogenic differentiation of amniotic fluid stem cells from both trimesters.** (A) Oil Red O staining (*left images*) (scale bar = 200 μm) and relative quantification (*right graph*) (expressed by ratio compared with control cells) (n = 5) performed at day 21 of differentiation. (B) Osteogenic differentiation was attained and calcium salt accumulation was detected by Von Kossa staining (*top left images*, brown-black deposits) (scale bar = 100 μm); calcium deposits from differentiating cells were also extracted and quantified at different time points (*top right graph*) (n = 3). Alkaline phosphatase activity was observed by colorimetric substrate modification (*bottom left images*) on differentiating cells at different time points. Alkaline phosphatase activity was also relatively quantified by measuring the substrate absorbance after reaction (*bottom right graph*) (expressed by ratio of differentiating over control cells, n = 3). ***P* < 0.01, ****P* < 0.001. *Ab* antibody, *ALP* alkaline phosphatase. (JPEG 8213 kb)

## Abbreviations

AcLDL: Acetylated-low density lipoprotein
AF: Amniotic fluid
AFS: Amniotic fluid stem
ALCAM: Activated leukocyte adhesion molecule
A.U.: Arbitrary units
CB: Cord blood
DAPI: 4′,6-diamidino-2-phenylindole
EGM-2: Endothelial growth medium-2
ETS: E-twenty six transformation
GFP: Green fluorescent protein
Hb: Hemoglobin
HUVEC: Human umbilical vein endothelial cell
LNGFR: Low-affinity nerve growth factor receptor
PBS: Phosphate-buffered saline
PCR: Polymerase chain reaction
PFA: Paraformaldehyde
qPCR: quantitative polymerase chain reaction
SCF: Stem cell factor
SDF-1: Stromal derived factor-1
SSEA4: Stage-specific embryonic antigen 4
VEGF: Vascular endothelial growth factor
vWF: von Willebrand factor
