# Supplementary figures and images for: Endothelial properties of third-trimester amniotic fluid stem cells cultured in hypoxia
Source: Stem Cell Res Ther. 2015 Oct 31;6:209. doi: 10.1186/s13287-015-0204-0 (PMC4628318; doi:10.1186/s13287-015-0204-0)

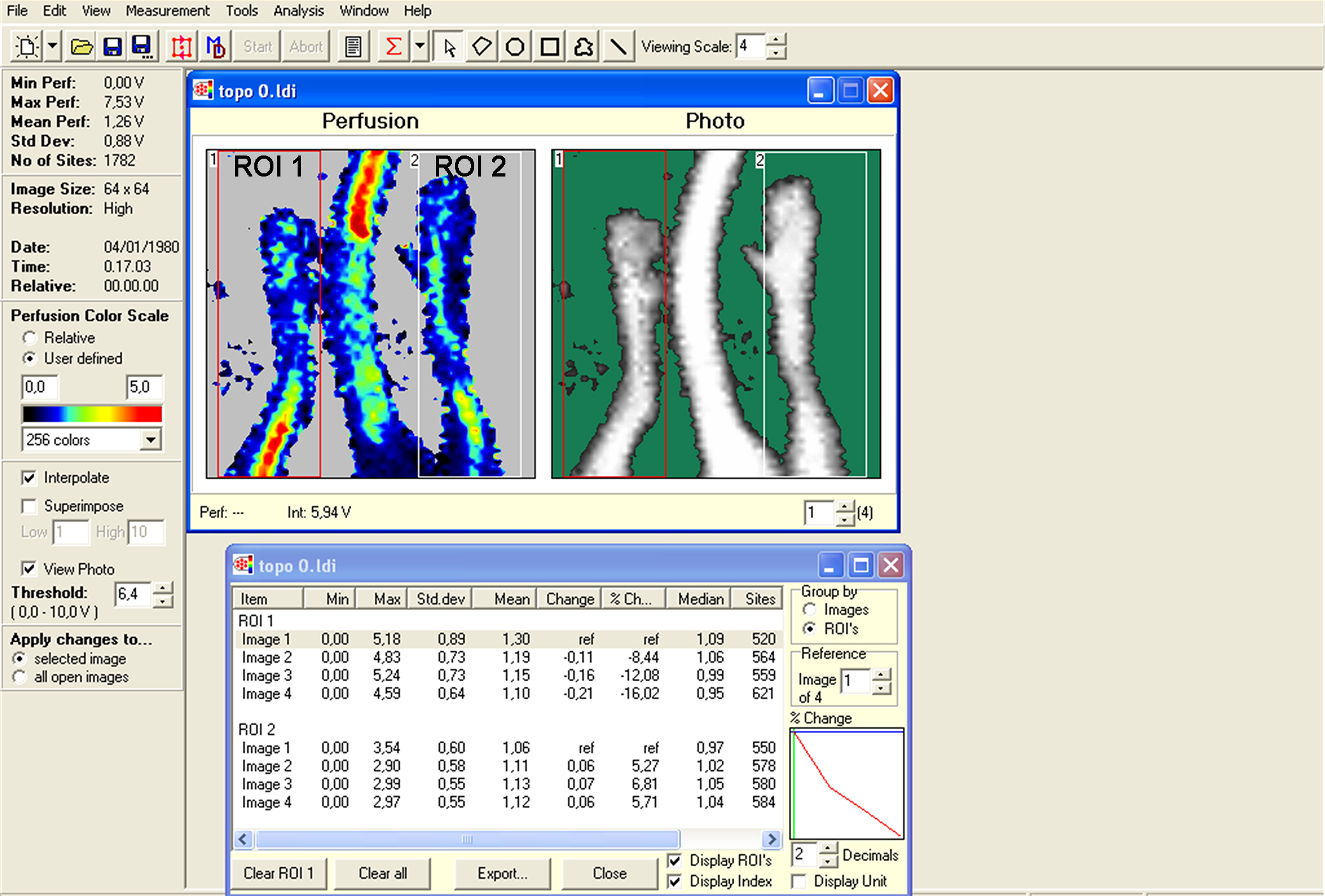

Supplement: Additional file 1: — Region of interest (ROI) visualization. Screen shot showing a representative image of an analyzed animal after ischemic damage and cell injection. The ischemic ratio was calculated by using ROI of the same size for the right and left limbs (ROI1 and ROI2). (JPEG 897 kb) [file 13287_2015_204_MOESM1_ESM.jpg]

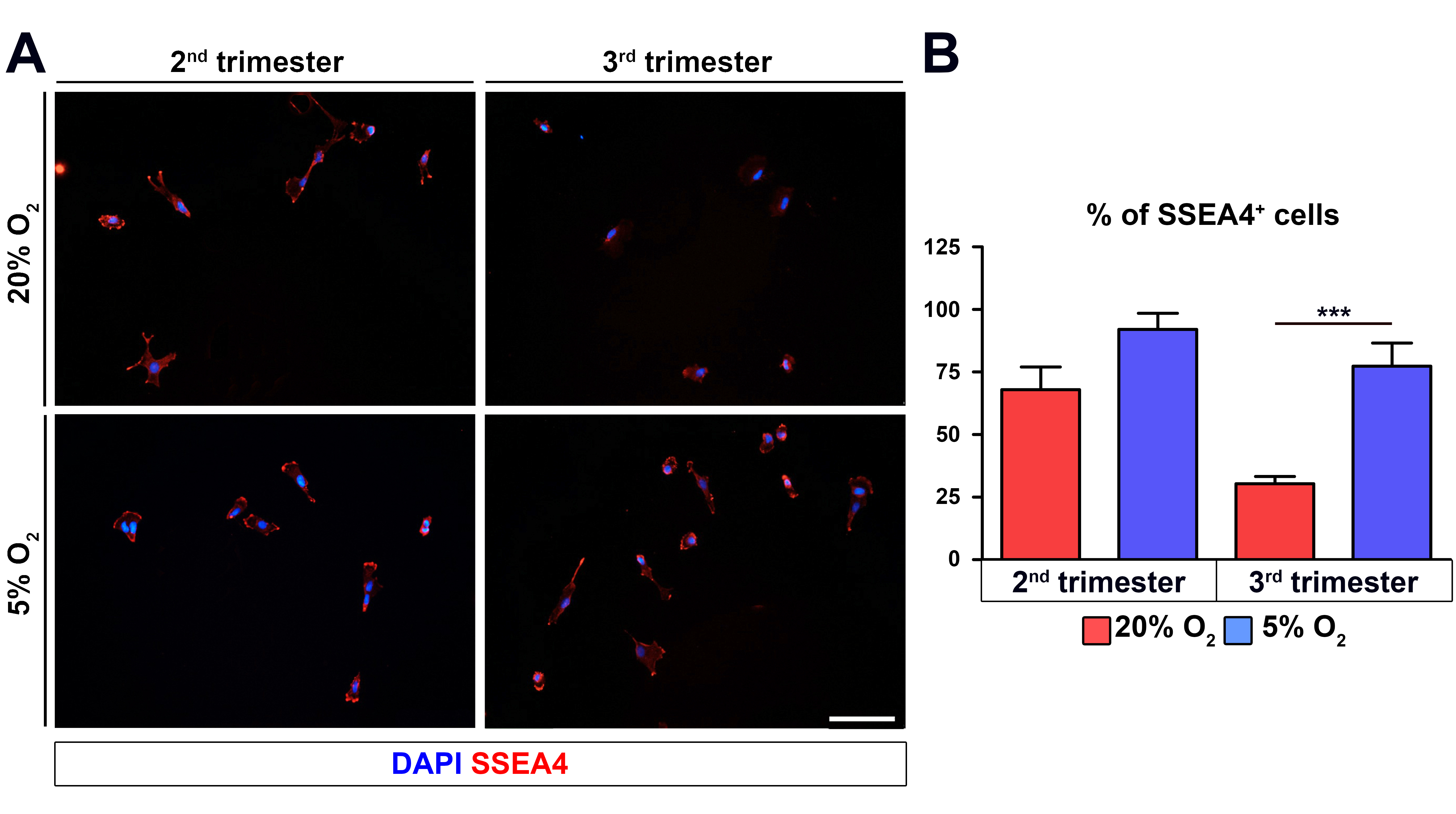

Supplement: Additional file 2: — Cell characterization. (A) SSEA-4 staining for AFS cells in all culture conditions. (B) Percentage of SSEA-4-positive AFS cells in all conditions. AFS amniotic fluid stem, SSEA-4 stage-specific embryonic antigen-4 (JPEG 2556 kb) [file 13287_2015_204_MOESM2_ESM.jpg]

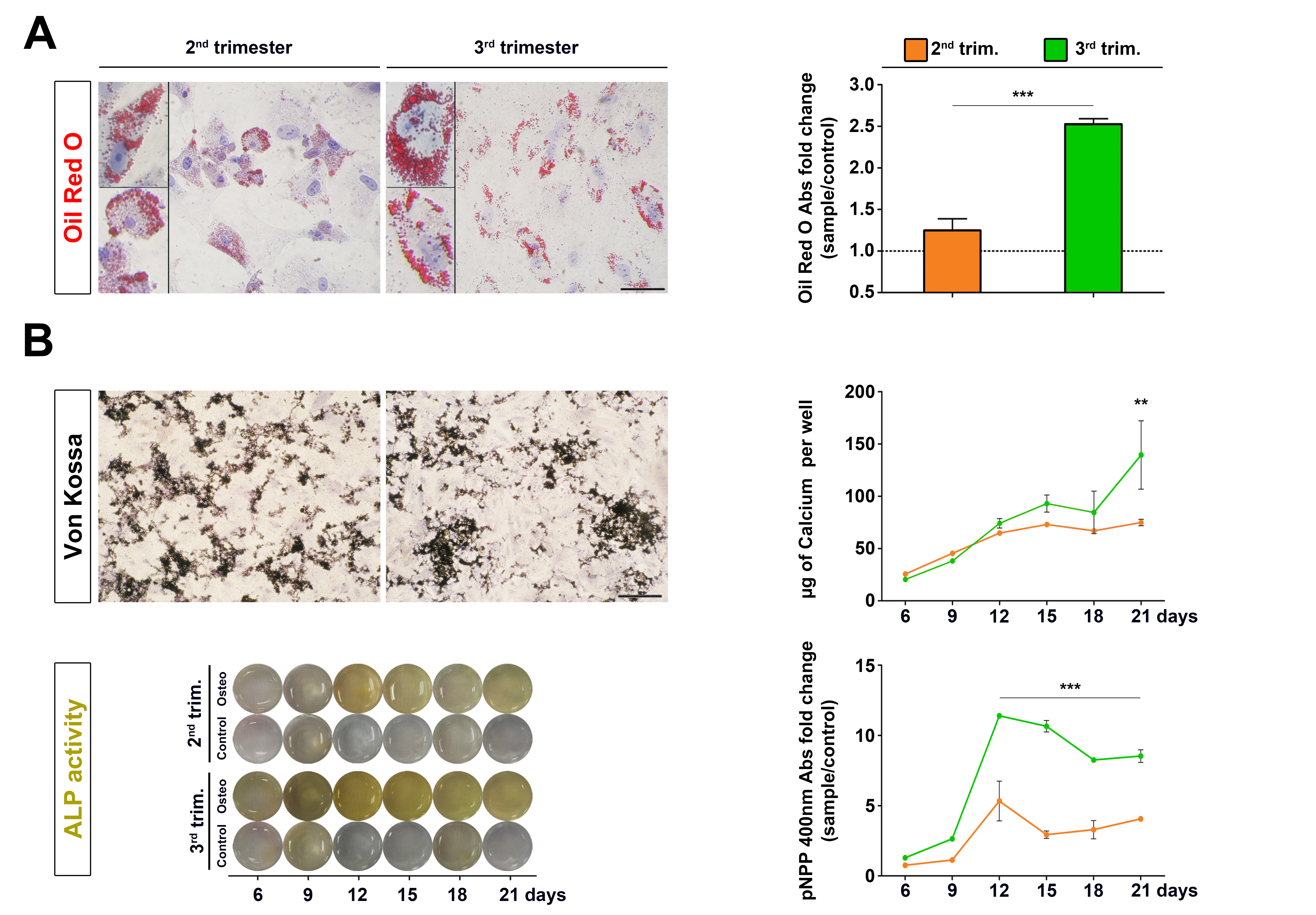

Supplement: Additional file 3: — Adipogenic and osteogenic differentiation of amniotic fluid stem cells from both trimesters. (A) Oil Red O staining (left images) (scale bar = 200 μm) and relative quantification (right graph) (expressed by ratio compared with control cells) (n = 5) performed at day 21 of differentiation. (B) Osteogenic differentiation was attained and calcium salt accumulation was detected by Von Kossa staining (top left images, brown-black deposits) (scale bar = 100 μm); calcium deposits from differentiating cells were also extracted and quantified at different time points (top right graph) (n = 3). Alkaline phosphatase activity was observed by colorimetric substrate modification (bottom left images) on differentiating cells at different time points. Alkaline phosphatase activity was also relatively quantified by measuring the substrate absorbance after reaction (bottom right graph) (expressed by ratio of differentiating over control cells, n = 3). **P < 0.01, ***P < 0.001. Ab antibody, ALP alkaline phosphatase. (JPEG 8213 kb) [file 13287_2015_204_MOESM3_ESM.jpg]
